# The Hospital Saving Scheme—Provincial Success and London Promise

**Published:** 1923-03

**Authors:** 


### THE HOSPITAL SAVING SCHEME.

### PROVINCIAL SUCCESS AND LONDON PROMISE.

At a recent meeting of the Hospital Officers' Association, Mr. F. B. Elliot, C.B.E., General Secretary of the Hospital Saving Association, delivered an interesting address upon the genesis and present position of the scheme. Mr. Elliot said that the scheme propounded by the Hospital Saving Association had been moulded by the Hospitals of London. It was still what it had originally been called, a "Hospitals of London Contributory Scheme." The method followed had been to circulate proposals to the London Hospitals, to submit them for discussion at the Regional Committee of the British Hospitals' Association, to invite criticisms and suggestions from all classes of hospital opinion and only to put a definite scheme before the public when the greatest common measure of agreement obtainable among hospitals had been secured.

### Provincial Contributory Schemes.

Provincial contributory schemes had been closely examined and the experience of those responsible for their control had been sought on points where difficulty was anticipated in London. What struck everyone who had looked closely into these Provincial schemes for organised collections of wage-earners' contributions was that, while they differed in detail in different centres, they had one common feature—their financial success and the stability which they had brought to the hospitals benefiting by them. In the returns issued by such hospitals there was a note of confidence and security which was lamentably absent from the literature generally issued by London hospitals. Glasgow could raise £90,000 a year in employees' contributions; at Newcastle almost half the income of the hospital was derived from the same source. Sheffield raised £41,000 in the first year of its contributory scheme, and looked forward to raising £100,000 to £150,000 annually. The Norfolk and Norwich hospital had experienced a large annual deficit, but in the second year of its contributory scheme income exceeded expenditure. It was not only that these schemes had brought an increased and stable income to the hospitals, but they had stimulated the interest of workpeople in the work of the hospitals, and had rooted them more firmly in their affections. The treasurer of the Oxford scheme had told him that in spite of the great fall in the wages of agricultural labourers there had been no sign of any desire to drop the weekly contribution to the hospital.

### Adaptation to London.

The scheme for London had been adapted to London conditions, both in form and in proposed administration. Wage-earners would be invited to form themselves into groups, primarily in their places of employment, for the purpose of making a regular voluntary contribution to the London hospitals. A standard contribution of 12s. per annum, paid in advance, or 3d. per week, had been laid down These contributions would be pooled and used for distribution to hospitals which had rendered services to contributors and their dependents. Contributors paying the standard contribution would not be required to make further payments if they or their wives or children found themselves in hospital. Contributors who would be drawn from the classes who now use the hospitals, would be admitted in accordance with the hospital's ordinary routine; the decision as to the suitability of the case for hospital treatment and the order of priority in which applicants, whether contributors or not, are admitted remains with the individual hospital. Group Secretaries would be supplied with a list of co-operating hospitals, and no doubt contributors would normally go to one of the hospitals which they knew would exempt them from Almoner's inquiries as to means and from requests for payment. If a contributor went to a non-co-operating hospital he would have no voucher, he would make his own terms with the hospital, pay himself, and, if the payment was a normal payment towards the cost of maintenance, he would subsequently get a refund through his Group Secretary.

### Work of the Central Organisation.

As to organisation, a central organisation had been set up with local branches. The Association would collect the contributions from the wage-earners on the one hand and would distribute them to hospitals on the other. Neither contributors nor hospitals would be members of the Association, but each would be fully represented on it. "Contributor Members" being men and women representative of the views of contributors, and "Hospital Members" nominated by hospitals, would each be entitled to elect one-third of the management. The remaining third would be drawn from employers, the King's Fund, and other central agencies, the medical profession, &c. The Provisional Executive Council was formed of men imbued with the voluntary spirit, and determined to preserve the autonomy and individuality of hospitals. They were anxious that the hospitals should take a full share in the work of the Association, both centrally and locally, and with this object in view, they had invited "key" hospitals to take the lead in initiating, with the co-operation of local hospitals in their area, branches of the Association.

### Immense Possibilities.

Competition between hospitals or between branches would be limited to healthy rivalry in recruiting contributors to the Association, and would not degenerate into squabbles over boundaries or competition in benefits under rival schemes, because contributions will be pooled and the same benefits offered to all contributors. They were anxious not to compete with the Hospital Saturday Fund, which for years had been collecting in the workshops of London; they desired to extend its activities and to settle the vexed questions which had arisen between the Fund and the hospitals since the system of patients' payments had been generally introduced. The Hospital Saturday Fund had already agreed to co-operate in principle, and he hoped before long that an arrangement would be made under which the Hospital Saturday Fund could, while retaining its identity and extending its utility, take an active and indeed prominent part in the Hospital Saving movement. The possibilities in London were, he was convinced, immense. He did not suggest that that income was going to roll in at once. It would take time and hard work to get the harvest in, but if they at the centre, and the hospital at the circumference, made up their minds to succeed, the voluntary system in London would be permanently established on a broad and sound financial basis.

### Still Room for Charity.

Charity would always be needed to provide for the indigent poor, and to extend the munificent work of the hospitals. What the Hospital Saving Association essayed to do was to relieve the hospitals of the cost of maintenance of patients in receipt of regular wages and small incomes. He believed that charity would be stimulated and the hospital subscriptions of employers particularly would be increased when it was found that those who use the hospitals and could afford to pay were paying. Under the scheme there was no interference with the voluntary system. Patients' payments at the present time, even when most vigorously pursued, did not, so far as he knew, bring in at any hospital more than an average of 18s. a week per in-patient. Whether or not the Association would be able at starting to pay the full cost of maintenance it would certainly be able to pay very much more than that. Though it might be advisable to spend a certain amount of working capital in general propaganda, he believed that the eventual scale of expense would be very small.

### The Teeth of Young Leeds.

Out of 29,000 Leeds school children referred to the dental clinics for treatment from January to November 11 of last year, only 12,000 accepted. It was in 1916 that the dental clinics were started. That they have not been without results is clear from the fact that in 1916 90 per cent. of the children required treatment for their teeth, while to-day the percentage is only 59.

